# Impact of Lockdown during COVID-19 Pandemic on Central Activation, Muscle Activity, Contractile Function, and Spasticity in People with Multiple Sclerosis

**DOI:** 10.1155/2021/2624860

**Published:** 2021-10-21

**Authors:** Luis Andreu-Caravaca, Domingo J. Ramos-Campo, Linda H. Chung, Pedro Manonelles, Oriol Abellán-Aynés, Jacobo Á. Rubio-Arias

**Affiliations:** ^1^International Chair of Sports Medicine, Catholic University of Murcia, Murcia, Spain; ^2^Faculty of Sport, Catholic University of Murcia, Murcia, Spain; ^3^LFE Research Group, Department of Health and Human Performance, Faculty of Physical Activity and Sport Science-INEF, Universidad Politécnica de Madrid, Madrid, Spain; ^4^UCAM Research Center for High Performance Sport, Catholic University of Murcia, Murcia, Spain; ^5^Department of Education, University of Almería, Almería, Spain

## Abstract

**Background:**

People with multiple sclerosis (MS) suffer from symptoms related to neural control, such as reduced central activation, lower muscle activity, and accentuated spasticity. A forced 9-week home confinement related to COVID-19 in Spain may have worsened these symptoms. However, no study has demonstrated the impact of home confinement on neuromuscular mechanisms in the MS population. This study was aimed at analyzing the effects of a 9-week home confinement on central activation, muscle activity, contractile function, and spasticity in MS patients.

**Methods:**

Eighteen participants were enrolled in the study. Left and right knee extensor maximum voluntary isometric contraction (MVIC), maximal neural drive via peak surface electromyography (EMG) of the vastus lateralis, central activation ratio (CAR), and muscle contractile function via electrical stimulation of the knee extensor muscles, as well as spasticity using the pendulum test, were measured immediately before and after home confinement.

**Results:**

Seventeen participants completed the study. CAR significantly decreased after lockdown (ES = 1.271, *p* < 0.001). Regarding spasticity, there was a trend to decrease in the number of oscillations (ES = 0.511, *p* = 0.059) and a significant decrease in the duration of oscillations (ES = 0.568, *p* = 0.038). Furthermore, in the left leg, there was a significant decrease in the first swing excursion (ES = 0.612, *p* = 0.027) and in the relaxation index (ES = 0.992, *p* = 0.001). Muscle contractile properties, MVIC, and EMG variables were not modified after confinement.

**Conclusions:**

The results suggest that a home confinement period of 9 weeks may lead to an increase in lower limb spasticity and a greater deficit in voluntary activation of the knee extensors.

## 1. Introduction

Multiple sclerosis (MS) is a clinically complex disease that affects the central nervous system [1]. Patients with MS show symptoms that mainly affect functional capacity [2] balance [3], and gait [4]. Pronounced muscle weakness, particularly in the lower limbs, can aggravate these symptoms in people with MS [5]. The mechanism underlying the deficit in muscle strength originates from both structural [6] and neural factors [7]. Regarding structural factors, different studies have found modifications in the distribution of muscle fibers [8] and muscle size [9] with respect to similar populations without diseases, while others have not found such changes [10]. Compared to healthy subjects, people with MS have lower cross-sectional area in both type I and II fibers [11]. This is mainly due to the lower rates of physical activity performed by people with MS [12], as well as the difficulty of the nervous system to recruit certain motor units. This inability to recruit certain motor units leads to their atrophy [13]. Lower limb muscles are more affected, as they are the ones most related to walking, mobility, and autonomy [2].

However, the central motor impairment largely explains the alteration in force production capacity in MS [7]. According to Ng et al. [7], people with MS present problems with motor unit recruitment, which leads to a higher deficit in voluntary muscle activation. This is measured using the central activation ratio (CAR), which provides information about the difference between the maximum voluntary force and the maximum force that would be produced if all motor units were recruited during a maximum contraction. Healthy people show a higher CAR than people with MS [7]. In this line, recent studies have shown that the maximum neural drive, measured through surface electromyography (EMG), is impaired in people with MS compared to people without clinical diseases [14]. In addition, a change in fiber-type distribution to faster and more glycolytic fibers after reduced activity has been observed in people with MS, resulting in an alternation in the muscle contractile properties [15]. A sedentary lifestyle and restricted movement could affect these changes in people with MS, leading to a decline in mobility, spasticity, and quality of life [16].

Along these lines, spasticity is another symptom that is prevalent in people with MS, and its occurrence is also due to changes in the central motor system. It is estimated that around 60% and 75% of the population with MS have high levels of spasticity [17]. Spasticity is caused by a lesion of the upper motor neuron and, consequently, leads to a nonsynchronous and intermittent activation of the muscles [18]. The presence of spasticity is related to poorer quality of life, as well as gait, mobility, and balance problems [19]. Spasticity is also worsened by a sedentary lifestyle [18].

Although neural and structural limitations elicit a variety of symptoms in people with MS, there are different treatments that can slow down their progress (e.g., pharmacological treatments and physical exercise training). In this context, physical exercise can be an effective nonpharmacological stimulus that can improve central activation [20, 21], muscle activity [22], and spasticity [23], among others. On the contrary, physical inactivity and sedentary lifestyle have been associated with lower levels of strength, greater central motor problems and higher levels of spasticity in people with MS with moderate and high disabilities [24]. Therefore, the 9-week home confinement related to the coronavirus disease-19 (COVID-19) pandemic in Spain has forced the general population to adopt an almost absolute sedentary lifestyle and sedentary behavior [25]. In a progressive disease, such as MS, where symptoms worsen over time, a sudden decrease in physical activity levels could aggravate these symptoms in a period of 9 weeks.

To our knowledge, this is the first study to demonstrate the impact of COVID-19-related home confinement on neuromuscular parameters in MS patients. Therefore, the aims of our study were (1) to analyze the effect of home confinement on central activation, muscle activity, and muscle contractile properties in people with MS and (2) to study whether home confinement had produced any increase in spasticity in this population.

## 2. Materials and Methods

### 2.1. Participants

Eighteen persons with MS participated in this study, and they were diagnosed with either Relapsing-Remitting or Primary Progressive MS by a board-certified neurologist using the McDonald criteria [26]. Participants were included if they were in the stable phase of the disease and were able to walk independently for more than 100 meters. Volunteers with MS were excluded from participation if they (1) did not have an expanded disability status scale between 2 and 6, (2) suffered a relapse within the prior 12 months, (3) were taking corticosteroid treatment within the prior 2 months, (4) were exercise training in the preceding 4 months, and (5) were participating in a home-based training program during home confinement. All participants gave signed, informed consent before starting the study.

### 2.2. Study Design

This study used a prospective observational cohort design. The participants visited the UCAM Research Center for High Performance Sport and UCAM Sport Center (Murcia, Spain) on 3 occasions. Testing sessions were performed at the same time of day to minimize differing responses due to circadian rhythm changes throughout the day. The first visit consisted of a familiarization session of all testing procedures. Subjects then returned 48 h later for the second visit to perform the neuromuscular and electromyographical assessments. Visit 3 took place 48 h later, where the spasticity measurement using the pendulum test was performed. This study was approved by the Science Ethics Committee of the Catholic University of Murcia in accordance to the Declaration of Helsinki [27]. The baseline measurements were conducted 5 days (March 1-5, 2020) before the National State of Alarm, obliging the entire population to home confinement for 9 weeks. These baseline measures were originally meant for an experimental intervention study, which was suspended due to the COVID-19 pandemic. When home confinement was no longer imposed, we felt it important to follow-up (i.e., postmeasurements) on these study participants. The postmeasurements were conducted 15 days after home confinement was lifted.

### 2.3. Testing Procedures

Prior to all tests that required the musculoskeletal system, a standardized warm-up was performed and consisted of 5 min on a cycle ergometer at 50 W and a dynamic stretching routine. Each assessment was conducted by the same researcher.

#### 2.3.1. Maximal Voluntary Isometric Contraction and EMG Measurements

Participants were seated upright on an isokinetic dynamometer chair (Biodex Medical System, NY) with the right knee flexed at 90° and the ankle fixed to a customized apparatus attached to a load cell (Model SML500, Interface Scottsdale, AZ, USA). Subjects performed 3 MVICs, each lasting for 5 s with 3 min of rest between contractions, and to ensure maximal performance, the highest 2 MVICs had to be within a 10% of difference. Participants were told to contract the muscle with “as much force as possible, as fast as possible.” Verbal encouragement was given throughout the contraction to ensure maximal effort. The highest trial was used for MVIC.

Surface EMG activity [28] was recorded from the vastus lateralis during the MVIC to assess neural drive. The skin was first prepared via shaving, abrasion, and cleansing with alcohol. Then, the upper electrode of each pair (Ambu Blue Sensor SP, Ambu A/S, Denmark) was positioned over the vastus lateralis following SENIAM Guidelines [29]. The exact electrode placements were carefully measured and mapped on transparent paper for subsequent measurements. The EMG activity from the vastus lateralis of the right leg were analyzed using the following time intervals: 0-30 ms (EMG 0-30), 0-50 ms (EMG 0-50), 0-100 ms (EMG 0-100), 0-200 ms (EMG 0-200), EMG Peak, and EMG time to peak. According to Aagaard et al. [28], EMG peak during maximal isometric contraction can be interpreted as maximal neural drive.

#### 2.3.2. Central Activation Ratio and Contractile Function

**Figure 1:**
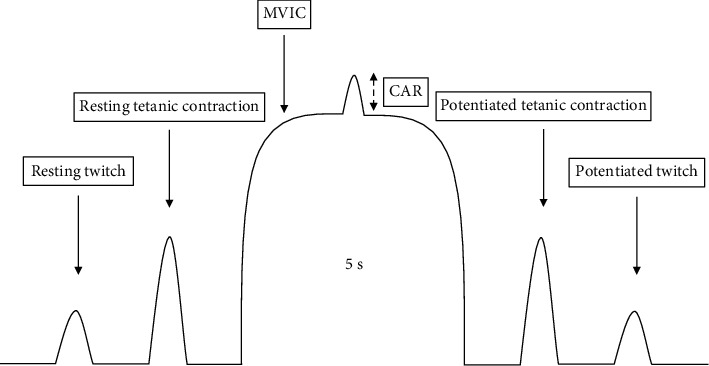
Illustration of the experimental protocol. CAR: central activation ratio; MVIC: maximal voluntary isometric contraction.

Two bipolar 10 × 15 cm stimulating electrodes were placed over the proximal and distal portions of the quadriceps of the right leg and secured with a Velcro wrap. Signal 6.0 software (CED, Cambridge, England) was used to control the electrical stimulation characteristics: 100 Hz, 50 pulses, length 0.009 s, and interval 0.01 s. The intensity of the stimulus was set at 40–50% of MVIC. To assess muscle contractile function and central activation ratio, participants underwent the following protocol (Figure 1): a single supramaximal stimulus (resting twitch); a 100 Hz, 50-pulse train of stimuli (resting tetanic contraction); an MVIC with a superimposed 100 Hz train when maximal force was steady; a 100 Hz, 50-pulse train of stimuli (potentiated tetanic contraction); and, finally, a single supramaximal stimulus (potentiated twitch). This sequence was repeated 2 times with 2 min of rest between measurements. Twitch-to-tetanus ratio (Tw/Tet) was calculated. Peak MVIC and peak force obtained by superimposed twitch and tetanus stimulation were determined. The CAR was calculated as follows: [30].

(1) $$\begin{array}{c} \text{CAR}=\frac{\text{MVIC}}{\text{MVIC}+\text{Superimposed train}}\cdot 100. \end{array}$$

#### 2.3.3. Spasticity

The participants were seated with the torso reclined approximately 30° (to avoid stretching of the biceps femoris) and the legs hanging freely over the edge of the seat. All subjects were barefoot and wore shorts. Three small circular markers were placed in the following anatomical positions in each leg: major trochanter of the femur, lateral epicondyle of the femur, and lateral malleolus of the fibula. A video was recorded during the pendulum test using a high-speed camera. Participants were instructed to close their eyes, remain silent, and keep their leg muscles completely relaxed. The researcher grasped the participant's heel and moved the leg from its resting position (~90° knee flexion) to full extension (~180° knee extension). The heel was then liberated to fall and oscillate until excursions came to a stop. The researcher determined that the participant maintained the leg at a relaxed state throughout the test by monitoring the absence of extraneous movement or muscle contraction. Two valid trials were performed with 30 seconds of rest between trials. The mean of the two trials was analyzed. The knee angles [31] were determined from the video recordings of each pendulum test (ImageJ software, version 1.42; National Institutes of Health, Bethesda, MD, USA). The first swing excursion was defined as the difference between the starting angle and the first inversion angle of the swinging limb. The starting angle was determined as the position at which the examiner released the participant's heel. The number of swings was established by counting the number of sine wave peaks produced by the swinging limb after the heel was released. The criterion for each oscillation was a shift of at least 3° towards extension. The duration (s) of the oscillations was also determined as the duration of the pendulum oscillations from the release of the lower limb to the end of the final oscillation, which was determined by the prior criterion. The relaxation index (RI) was calculated as follows: RI = (initial angle − first angle)/(initial angle − rest angle). The rest angle was the position of the knee joint that was maintained after the oscillatory movement ceased.

### 2.4. Statistical Analyses

Data collection, treatment, and analysis were performed using the SPSS for Windows statistical package (version 20.0; SPSS, Inc., Chicago, IL, USA). Descriptive statistics (mean and SD) were calculated. Before using parametric tests, the assumption of normality was confirmed with the Shapiro-Wilks test. Student's *t*-test for pair samples was used to test if significant changes occurred pre- and posthome confinement. A level of *p* ≤ 0.05 was set to indicate statistical significance. The effect size (ES) was calculated using Cohen's guidelines [32], and the Cohen scale was used to demarcate effect sizes, where 0.2 represents a small effect, 0.5 a moderate effect, and 0.8 a large effect.

## 3. Results

Seventeen participants with MS completed the study (one participant dropped out due to schedule incompatibility with the posttesting sessions).  Table 1 [33] shows the participant characteristics. Participants reported that they had not undertaken any home training programs.

### 3.1. Maximal Voluntary Isometric Contraction and EMG Measurements

**Figure 2:**
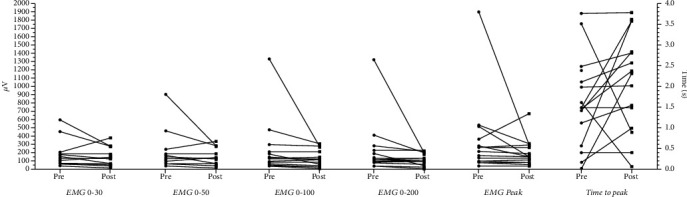
Effects of home confinement on muscle activity during knee extension MVIC. EMG: surface electromyography; MVIC: maximal voluntary isometric contraction.

EMG time-to-peak (s) tended to increase with a moderate effect after home confinement (*d* = −0.526, *p* = 0.061) (Figure 2). No changes were observed in the other EMG variables.

### 3.2. Central Activation Ratio and Contractile Function

A significant decrease with a large effect size was observed in CAR after home confinement (*d* = 1.271, *p* < 0.001). No changes were observed in any of the analyzed contractile function variables (resting twitch, resting tetanic contraction, 1st Tw/Tet, potentiated twitch, potentiated tetanic contraction, and 2nd Tw/Tet; *p* = 0.088) (Table 2).

### 3.3. Spasticity

On the right leg, a significant reduction with a moderate effect was observed in the oscillation duration after home confinement (*d* = 0.568, *p* = 0.038). In addition, number of oscillations tended to decrease with a moderate ES (number of oscillations: *d* = 0.511, *p* = 0.059) at posthome confinement.

On the left leg, a significant decrease was observed in the first swing excursion (*d* = 0.612, *p* = 0.027). Furthermore, RI significantly decreased in post measurements (*d* = 0.992, *p* = 0.001) (Table 3). No changes were found in the other spasticity variables.

## 4. Discussion

Our study was aimed at analyzing the impact of home confinement due to the COVID-19 pandemic on central activation, muscle activity, muscle contractile function, and spasticity in people with MS. The results showed that voluntary muscle activation decreased after home confinement, which was explained by the lower central activation of the muscle. In addition, home confinement caused an increase in spasticity in our sample population with MS.

### 4.1. Effects of Home Confinement on Neuromuscular Mechanisms

MVIC showed no statistically significant change after home confinement. The deficits in maximal strength of the lower limb muscles that are present in this population [5] can lead to significant problems, such as impaired gait kinematics [34], poor postural control [35], and reduced functional capacity [2]. Although the changes were not significant, our study showed a mean decrease of 5% in lower body maximal strength following 9 weeks of home confinement. This decrease may be explained mainly by impairments in the neural component of the neuromuscular system, where decreases in CAR and EMG peak and increases in spasticity were observed. In this context, the muscle activity of the vastus lateralis (EMG) of all of the analyzed time intervals decreased, but not significantly, during knee extensor maximal isometric contraction at posthome confinement. In addition, maximal neural drive (EMG peak) showed a small effect size, but it was not statistically significant. Previous studies found that people with MS had 54% less vastus lateralis activity (surface EMG) during MVIC compared to control subjects [14]. Since there is an existing deficit in muscle activity in people with MS, a drastic decrease in physical activity, such as that imposed by forced home confinement, would result in a greater deficit in neuromuscular activity. In general, lower muscle activity in MS patients can be explained by their inability to activate all motor neurons of the vastus lateralis [7, 15], as well as having lower motor unit firing rates [36]. Thus, resistance training may be a promising approach to reverse these neuromuscular changes. A previous study by Dalgas et al. [20] demonstrated that 12 weeks of resistance training improved neural drive (EMG peak) in people with MS. These data indicate that neural plasticity in response to resistance training is preserved in people with MS, despite the impairment of MS in the central nervous system [20]. Although EMG activity is a valid tool to measure neural drive, other variables, such as fiber size, fiber-type composition, intramuscular ionic concentrations, and sodium-potassium pump content [37], could have been altered after 9 weeks of home confinement.

In addition, CAR decreased significantly (pre vs. post -2.8%) after the period of home confinement. A decrease in CAR implies a greater voluntary muscle activation deficit. Previous studies affirm that CAR is lower in people with MS than in the population without disease [7]. In this context, it has been established that resistance training has the capacity to increase CAR [38, 39]. The mechanisms that have been suggested to improve CAR are better synchronization and motor unit recruitment [7]. Additionally, decreased antagonist muscle activity during agonistic muscle contraction and increased corticospinal excitability are factors that may contribute to increases in CAR [40]. Although there are no previous studies analyzing the consequences of physical inactivity or detraining period in CAR in people with MS, our study observed that COVID-19-related sedentary behavior (e.g., increased sitting time and decreased number of daily steps) during home confinement had a profound impact on voluntary muscle activation in people with MS. The decrease in CAR leads to a decline in maximal force production and diminished capacity to perform explosive actions in short periods of time [41]. Thus, variables related to maximal muscle strength or rate of force development, such as balance [35], mobility [42], gait [34], and spasticity [43], may be impaired.

No changes were found in pre- and post-MVIC twitch and tetanic forces after 9 weeks of home confinement. The lack of change in twitch and tetanic forces suggest that the changes in MVIC are mainly explained by lower central activation and not due to alterations in the peripheral contractile properties. These findings agree with those found in previous investigations that showed that neural changes, measured through neural drive and central activation, are more important in strength reductions [7], although a period of inactivity can interfere with the contractile properties of the muscle. Perhaps longer or more severe periods of physical inactivity (such as bed rest) might have significantly affected the contractile properties of the muscle fibers [44].

### 4.2. Effects of Home Confinement on Spasticity

The findings of this study shed light on the effects of decreased physical activity on spasticity, one of the most prevalent symptoms in MS. Although previous studies had established a strong relationship between physical activity and spasticity in this population [18], no study has had the opportunity to examine the effects of home confinement, with its associated consequences, on this variable. In our study, both the number and the duration of oscillations (right leg), as well as the first swing excursion and relaxation index (left leg), decreased after home confinement, indicating increased spasticity. High levels of spasticity generate gait problems [45], increased energy cost of walking [46], poor balance [47], reduced mobility [19], and, consequently, poorer quality of life [48]. From a neuromuscular perspective, increases in spasticity may be due to worsening of synchronization and recruitment of the motor units [18], along with an augmented resistance to muscle lengthening due to the activation of tonic stretch reflexes [24]. This could also be associated with the significant decreases in CAR, suggesting that home confinement impairs neural control. Moreover, spasticity is a multifactorial problem, which is also influenced by psychological and behavioral aspects. The psychological and behavioral impacts of the health emergency caused by COVID-19 (e.g., fear of infection, anxiety [49], and poorer sleep quality [50]) may have contributed to increased spasticity in people with MS [51].

## 5. Limitations of the Study

The study has some limitations, and therefore, the results should be interpreted with caution. The sample population was composed of men and women with different MS phenotypes, which may have affected the results. In addition, we did not objectively quantify the level of physical activity (i.e., with accelerometers) during the 9 weeks of home confinement, and thus, we cannot confirm that the participants did not perform any home training program, although the study criteria included only those who did not perform any exercise training. In addition, electrical stimulation was not performed over the nerve but over the muscle. Although the method used in our study has been extensively validated, it may present some difficulties in the interpretation of the results. Furthermore, the force data in the different measured intervals of the EMG were not analyzed. Finally, we did not have a high-density electromyography decomposition to indirectly estimate motor unit firing rates.

## 6. Conclusion

The present study shows the adverse consequences of 9 weeks of home confinement on voluntary activation and spasticity in persons with moderate MS disability. In a progressive disease such as MS in which pharmacological and nonpharmacological treatments are aimed at slowing down the symptoms and disability of the disease, a significant worsening of neural activation and an increase in spasticity observed in this study highlights the risk that physical inactivity and sedentary behavior have on this MS population. These results provide evidence for the need to implement home training programs in the daily rehabilitation of people with MS. Due to the duration of confinement (9 weeks), our results suggest that the adverse effects were greatest on the neural component.

## Figures and Tables

**Table 1:** Participant characteristics [33].

Characteristics	Mean ± SD (*n* = 17)
Age (yrs)	43.50 ± 11.23
Sex (men : women)	7 : 10
EDSS (a.u.)	2.87 ± 1.38
Type of MS (RRMS : SPMS)	15 : 2
Weight (kg)	70.63 ± 12.34
Height (cm)	167.69 ± 7.18
Lean mass (kg)	51.70 ± 9.56
Fat mass (%)	27.46 ± 9.80
BMI (kg/m^2^)	25.01 ± 3.36

Values are means ± SD. BMI: body mass index; EDSS: expanded disability status scale; MS: multiple sclerosis; RRMS: relapsing-remitting multiple sclerosis; SD: standard deviation; SPMS: secondary progressive multiple sclerosis.

**Table 2:** Comparison pre-post effect on neuromuscular characteristics.

Muscle strength	Pre (mean ± SD)	Post (mean ± SD)	Δ ± ΔSD	*t*	*p*	Effect size	95% CI for Cohen's *d*	
							Lower	Upper
MVIC (*N*)	446.0 ± 196.0	424.0 ± 162.0	−0.03 ± 0.09	1.774	0.096	0.443	-0.078	0.952
*Stimulated force*								
Resting twitch (*N*)	25.0 ± 14.4	25.5 ± 15.5	0.06 ± 0.62	-0.613	0.872	-0.040	-0.530	0.450
Resting tetanic contraction (*N*)	259.0 ± 103.0	277.0 ± 94.1	0.15 ± 0.35	-1.252	0.230	-0.313	-0.811	0.194
1^st^ Tw/Tet	0.10 ± 0.05	0.09 ± 0.06	−0.07 ± 0.44	0.592	0.562	0.148	-0.347	0.638
Potentiated tetanic contraction (*N*)	247.0 ± 81.1	230.0 ± 69.1	−0.05 ± 0.16	1.822	0.088	0.456	-0.067	0.965
Resting twitch (*N*)	33.8 ± 17.6	36.4 ± 17.8	0.21 ± 0.77	-0.796	0.439	-0.199	-0.691	0.299
2^nd^ Tw/Tet	0.13 ± 0.05	0.16 ± 0.08	0.27 ± 0.72	-1.314	0.209	-0.329	-0.827	0.180
*Central activation*								
CAR (%)	91.5 ± 5.3	88.7 ± 4.2	−0.03 ± 0.02	5.084	<0.001	1.271	0.594	1.925

Values are means ± SD. CAR: central activation ratio; EMG: electromyography; MVIC: maximal voluntary isometric contraction; SD: standard deviation; Tw/Tet: twitch-to-tetanus ratio.

**Table 3:** Comparison pre-post effect on spasticity.

	Spasticity	Pre (mean ± SD)	Post (mean ± SD)	Δ ± ΔSD	*t*	*p*	Effect size	95% CI for Cohen's *d*	
								Lower	Upper
Right leg	First swing excursion (°)	95.6 ± 18.7	88.2 ± 17.6	−0.06 ± 0.23	1.910	0.075	0.477	-0.048	0.989
	Number of oscillations (*n*)	11.6 ± 4.3	10.6 ± 3.8	−0.05 ± 0.19	2.043	0.059	0.511	-0.019	1.025
	Duration of oscillations (s)	7.6 ± 2.3	6.7 ± 2.4	−0.10 ± 0.18	2.271	0.038	0.568	0.030	1.089
	Relaxation index	1.6 ± 0.4	1.7 ± 0.3	0.21 ± 0.54	-1.563	0.139	-0.391	-0.893	0.125
Left leg	First swing excursion (°)	96.6 ± 21.1	89.0 ± 15.5	−0.06 ± 0.13	2.448	0.027	0.612	0.068	1.139
	Number of oscillations (*n*)	11.3 ± 3.4	10.6 ± 3.8	−0.04 ± 0.28	1.180	0.256	0.295	-0.211	0.791
	Duration of oscillations (s)	7.1 ± 1.8	6.5 ± 2.5	−0.11 ± 0.21	1.725	0.105	0.431	-0.089	0.938
	Relaxation index	1.7 ± 0.2	1.6 ± 0.3	0.11 ± 0.11	3.967	0.001	0.992	-1.584	-0.378

Values are means ± SD. SD: standard deviation.

## Data Availability

The data that support the findings of this study are available from the corresponding author upon reasonable request.
